# State-of-the-Science of human papillomavirus vaccination in women with human immunodeficiency Virus: Summary of a scientific workshop

**DOI:** 10.1016/j.pmedr.2023.102331

**Published:** 2023-07-19

**Authors:** Anne E. Schuind, Helen Rees, John Schiller, Nelly Mugo, Peter Dull, Ruanne Barnabas, Gary M. Clifford, Gui Liu, Shabir A. Madhi, Rebecca B. Morse, Anna-Barbara Moscicki, Joel M. Palefsky, Stanley Plotkin, Mónica S. Sierra, Mark K. Slifka, Alex Vorsters, Aimée R. Kreimer, Arnaud M. Didierlaurent

**Affiliations:** a PATH, Washington DC, United States; b Wits Reproductive Health and HIV Institute (Wits RHI), University of the Witwatersrand, Johannesburg, South Africa; c National Cancer Institute, National Institutes of Health, Bethesda, United States; d Kenya Medical Research Institute, Nairobi, Kenya and Department of Global Health, University of Washington, Seattle, United States; e Vaccine Development, Bill & Melinda Gates Foundation, Seattle, United States; f Division of Infectious Diseases, Mass General Hospital, Harvard Medical School, Boston, United States; g Early Detection, Prevention, and Infections Branch, International Agency for Research on Cancer (IARC/WHO), Lyon, France; h Department of Global Health, University of Washington, Seattle, United States; i South African Medical Research Council Vaccines and Infectious Diseases Analytics Research Unit, Johannesburg, South Africa; j Centre of Vaccinology, University of Geneva, Switzerland; k Department of Pediatrics, University of California Los Angeles, Los Angeles, United States; l University of California, San Francisco School of Medicine, San Francisco, United States; m Department of Pediatrics, Perelman School of Medicine, University of Pennsylvania, Philadelphia, United States; n Oregon Health & Science University, Beaverton, United States; o Vaccine & Infectious Disease Institute, Centre for the Evaluation of Vaccination, University of Antwerp, Antwerp, Belgium

**Keywords:** Human papillomavirus (HPV), human immunodeficiency virus (HIV), women with HIV (WWH), Epidemiology, Immunology, HPV vaccine, Prevention, Vaccination strategies, Immunization

## Abstract

The burden of cervical cancer is disproportionately distributed globally, with the vast majority of cases occurring in low- and middle-income countries. Women with human immunodeficiency virus (HIV) (WWH) are at increased risk of human papillomavirus (HPV) infection and cervical cancer as compared to HIV-negative individuals. HPV vaccination remains a priority in regions with a high burden of cervical cancer and high HIV prevalence. With HPV vaccines becoming more accessible, optimal use beyond the initial World Health Organization-recommended target population of 9 to 14-year-old girls is an important question. In March 2022, a group of experts in epidemiology, immunology, and vaccinology convened to discuss the state-of-the-science of HPV vaccination in WWH. This report summarizes the proceedings: review of HIV epidemiology and its intersection with cervical cancer burden, immunology, HPV vaccination including reduced-dose schedules and experience with other vaccines in people with HIV (PWH), HPV vaccination strategies and knowledge gaps, and outstanding research questions. Studies of HPV vaccine effectiveness among WWH, including duration of protection, are limited. Until data from ongoing research is available, the current recommendation for WWH remains for a multi-dose HPV vaccination regimen. A focus of the discussion included the potential impact of HIV acquisition following HPV vaccination. With no data currently existing for HPV vaccines and limited information from non-HPV vaccines, this question requires further research. Implementation research on optimal HPV vaccine delivery approaches for WWH and other priority populations is also urgently needed.

## Highlights

• HPV vaccination is a priority in regions with high burden of cervical cancer and HIV.
• Research needs to address level and duration of protection for HPV vaccines in WWH.
• The effect of later HIV acquisition on HPV vaccine effectiveness is not understood.
• Catchup vaccination could substantially reduce cervical cancer incidence in WWH.
• Research is needed on HPV vaccine delivery to WWH and other at-risk populations.

## 1 Introduction

Persistent infection with oncogenic human papillomavirus (HPV) types is a necessary antecedent condition for cervical cancer. Cervical cancer is the fourth most common cancer among women globally, with 604,127 new cases and 341,831 deaths in 2020 (Ferlay et al., 2020). Cervical cancer remains a disease of geographic and economic inequality, with nearly 90% of the deaths occurring in low-and middle-income countries (Bruni et al., 2021a).

HPV infections are very common and in particular among people with compromised immune systems (e.g., people with human immunodeficiency virus (HIV) (PWH), organ transplants, or autoimmune diseases). Sub-Saharan Africa (SSA) carries the highest burden of HIV and cervical cancer as compared to the rest of the world (Ibrahim Khalil et al., 2022). Currently, there are four licensed, highly effective, and safe virus-like particle (VLP)-based HPV vaccines that have obtained the World Health Organization’s (WHO) Prequalification. Merck’s quadrivalent (Gardasil®) and nonavalent (Gardasil®9) vaccines and GlaxoSmithKline’s bivalent (Cervarix®) vaccine are currently recommended by WHO as either a 1- or 2-dose regimen in 9 to 14-year-olds (routine immunization) and up to age 20 (catch-up programs). The fourth licensed and prequalified vaccine, Innovax’s bivalent (Cecolin®) vaccine, is recommended as a 2- or 3-dose regimen for girls 9 to 14 and a 3-dose regimen for women above 14 years of age. Until further evidence is available, WHO recommends that immunocompromised persons 9 years and older ideally receive 3 doses but at least 2 doses of HPV vaccine (World Health Organization, 2022). Licensure trials did not include PWH; thus recommendations are based on the potential for immune dysregulation to decrease vaccine immunogenicity.

Despite HPV vaccines having been available for > 15 years, less than a third of the global population of girls live in countries with HPV vaccination programs, leading to a global coverage of 13% in girls below 15 years being fully vaccinated (World Health Organization, 2020b). In August 2020, the World Health Assembly adopted a global strategy to accelerate the elimination of cervical cancer as a global health problem (World Health Organization, 2020a). One of the strategy’s three foundational pillars is to achieve HPV vaccination in 90% of girls by 15 years of age by 2030.

In March 2022, a scientific workshop convened experts in epidemiology, immunology, and vaccinology to discuss the state-of-the-science of HPV vaccination in women with HIV (WWH). Two questions were posed to the experts: what is the performance of HPV vaccination among people who have HIV infection at the time of vaccination, and does HIV acquisition after HPV vaccination of young girls negatively impact vaccine-mediated protection? The experts also discussed the number of HPV vaccine doses required in these two scenarios.

This manuscript summarizes the meeting proceedings, including the intersection between HIV and HPV epidemiology, the potential impact of HIV infection on vaccine immunology, experience with HPV vaccines and other vaccines in PWH, and HPV vaccination strategies in the context of high HIV prevalence. The panel also discussed key knowledge gaps and outstanding research questions.

## 2 Global HIV epidemiology and intersection with cervical cancer burden

WWH have six times the risk of developing cervical cancer compared to women without HIV infection (Stelzle et al., 2021). In SSA, the region that bears the highest HIV-attributable cervical cancer burden, incidence rates in WWH in the era of antiretroviral therapy (ART) reach levels of > 200 cases per 100,000 person-years (Dhokotera et al., 2019; Rohner et al., 2020). This excess risk is driven by a combination of four factors: 1) high prevalence of HPV in settings with moderate to high HIV prevalence 2) increased cervical HPV acquisition due to shared transmission routes of HIV and HPV, 3) acceleration of HPV carcinogenesis due to HIV-related immunosuppression (increased risk of cervical HPV persistence and progression to (pre)cancer), and 4) limited access to high-quality prevention and treatment programs.

HIV incidence increases between ages 15 and 19 in most SSA populations, reflecting mean ages of sexual debut (Akullian et al., 2021, Risher et al., 2021). HPV incidence is more difficult to capture than for HIV but occurs shortly after initiation of sexual activity and is expected to follow a similar age-specific pattern in these settings (de Sanjosé et al., 2007, Risher et al., 2021). The role of perinatal HPV infection among those with perinatal HIV is unknown, should HPV also be transmitted from mother to infant along with HIV. Globally, the contribution of HIV to cervical cancer is highest in those cases diagnosed in younger women (Ibrahim Khalil et al., 2022).

With respect to HPV natural history, HIV infection increases risk of HPV persistence and progression to cervical pre-cancer. However, these risks are associated with lower CD4^+^ cell counts and can be partly reduced by timely access to ART (Kelly et al., 2018, Liu et al., 2018). Thus, increased and early access to ART is expected to lead to a decline in the incidence rate of cervical cancer among WWH (Liu et al., 2022).

Further, numbers of new HIV infections are steadily declining including in girls aged 15 to 19 years (Joint United Nations Programme on HIV/AIDS, 2020) in SSA, from 188,000 new cases in 2010 to 138,000 in 2020, thanks to improving HIV control measures. Consequently, increasingly fewer girls aged 15 to 19 years are predicted to be living with sexually acquired HIV (Risher et al., 2021), ultimately leading to a decline in HIV-attributable cervical cancer. Cohorts of perinatally infected WWH who are under long-term treatment and surveillance are also now attaining 15 years of age in SSA (Zanoni et al., 2016), but they are not usually distinguishable from sexually infected WWH in routine HIV prevalence statistics. HIV prevalence estimates in 15 to 19-year-olds are currently highest in Eswatini, Lesotho, South Africa, and Mozambique, ranging from 6% to 7% (Joint United Nations Programme on HIV/AIDS, 2020).

HPV vaccination remains a priority in regions with a high burden of cervical cancer and high HIV prevalence, particularly since cervical cancer screening programs are suboptimal in these regions. The above data highlight the major public health relevance of widespread HPV vaccination prior to sexual debut in settings with high burden of both HIV and HPV.

## 3 Impact of HIV infection on HPV and other vaccinations

### 3.1 Immune dysregulation associated with HIV infection and potential impact on HPV vaccine response

Acute and chronic HIV infection leads to dysregulation of the immune system due to extensive immune activation and chronic inflammation, impacting immune cells involved in the initiation and maintenance of vaccine-induced immunity (Appay et al., 2019, El Chaer and El Sahly, 2019). This results in a reduced response to most vaccines in PWH (Kernéis et al., 2014). With the use of ART early after infection, the long-term effects of HIV acquisition on immune cells have been significantly reduced but not completely abrogated (Chiappini et al., 2018, Zicari et al., 2019), which may explain why reduced response to vaccines is observed in children with HIV (CWH) despite ART (Abzug et al., 2009).

**Fig. 1:**
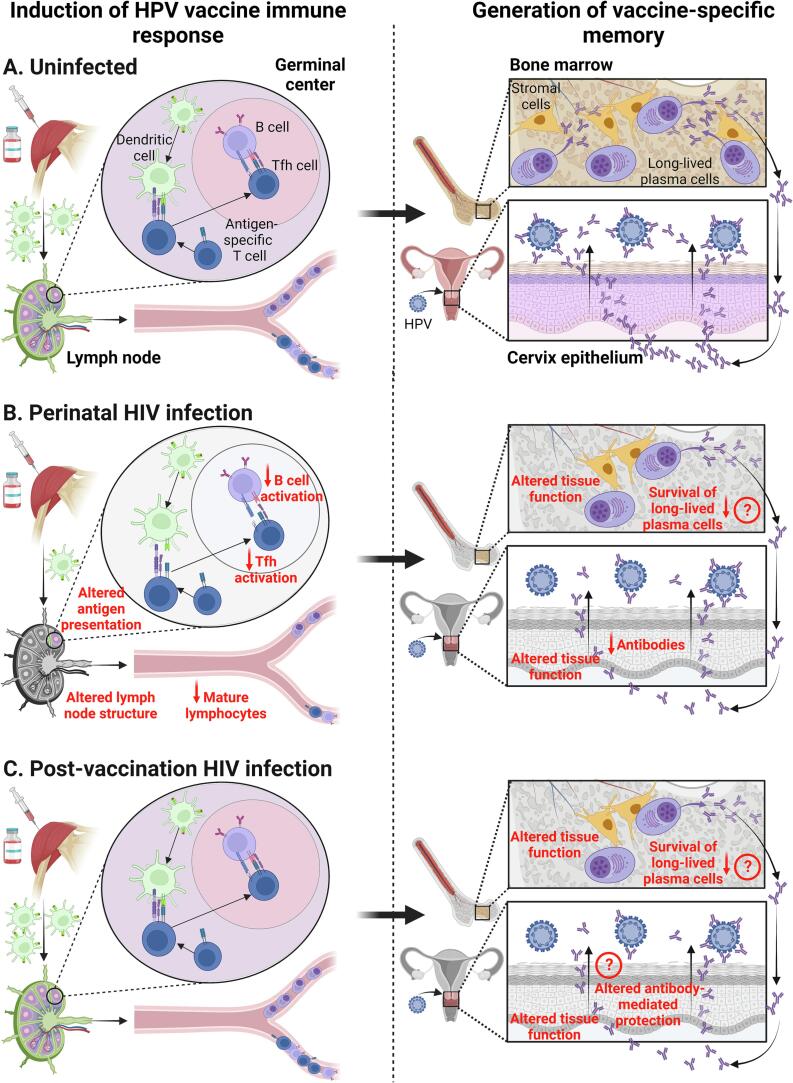
Potential impact of the immune dysregulation associated with HIV infection on HPV vaccine response. Following immunization with HPV vaccine, antigen processing and presentation to cognate T cells occurs in the lymph node draining the injection site. In the germinal center of the lymph node, the interaction between antigen-specific T follicular helper (Tfh) cells and B cells generates memory B cells and antibody-producing plasma cells. Long-lived plasma cells migrate to the bone marrow and produce a sustained level of HPV-specific antibodies able to prevent infection at the cervix epithelium (A). In people perinatally infected with HIV (B), alteration of the lymph node structure as well as an impairment in antigen presentation and activation of B cells and Tfh cells may persist despite early antiretroviral therapy. This may eventually reduce vaccine-induced antibody responses and long-lived plasma cell generation. HIV infection is also thought to impact the survival of long-lived plasma cells in the bone marrow, which may further reduce antibody titers, notably in those who acquire HIV after vaccination (C). It is also unclear whether HIV could alter the cervical tissue and affect the function or transudation of HIV-specific antibodies, thereby potentially reducing protection against HPV infection. Created with BioRender.com.

Following intramuscular immunization with HPV vaccine, antigen processing and presentation to cognate T cells occur in the lymph node draining the injection site (Fig. 1). The interaction between antigen-specific T cells and B cells in specific areas of the lymph node generates memory B cells and antibody-producing plasma cells. Long-lived plasma cells migrate to the bone marrow and produce a sustained level of HPV-specific antibodies able to prevent infection at the cervix epithelium. Some of the immune cells involved in HPV vaccine response may be altered in PWH.

The question remains whether the HPV vaccine will be sufficiently immunogenic in individuals infected perinatally or who recently acquired HIV before or between doses, particularly in settings where ART initiation is delayed or adherence to ART is variable. Perinatally HIV-infected children have chronic persistent inflammation (Zicari et al., 2019). Signs of premature immunosenescence (lymphopenia, reduced progenitors) are observed in young PWH despite a normalized CD4^+^ T cell count (Fastenackels et al., 2019). Persistent disruption of the lymph node infrastructure (Sanchez et al., 2015) and altered number and function of antigen-presenting cells, such as dendritic cells (Murray et al., 2020) or monocytes (Espíndola et al., 2018), have also been reported; however, whether these sustained defects affect vaccine response is unclear. In a cohort of perinatally infected children comparing treated and untreated patients, the number of memory B cells (cells that are recalled to proliferate and produce antibodies upon booster vaccination or re-exposure to pathogen) was not completely restored by ART (McCarty et al., 2018), although this could improve through early initiation of ART (Shalekoff et al., 2021). Reduced function of T follicular helper cells, which are critical for the differentiation of B cells into high-affinity antibody producers, correlates with poor response to an influenza vaccine in CWH who had undetectable HIV-1 virus (de Armas et al., 2021). However, in CWH on antiretroviral treatment, the number of T follicular helper cells is partially restored (McCarty et al., 2018). Altogether, these data show that a residual impairment in B and T cells persists in ART-treated CWH and that this is consistent with the observed decreased immune responses to HPV vaccines in PWH (Lacey, 2019).

Data are limited on whether HIV acquisition after HPV vaccination could impair the durability of the antibody response. Antibody titers to pneumococcal and tetanus vaccines given during childhood are reduced in adults who subsequently became infected with HIV, irrespective of ART, as compared to uninfected controls (Hart et al., 2007). Further, 20% of PWH gradually lose detectable antibodies to a vaccinia vaccine received during childhood, although they are maintained in HIV-negative vaccinated individuals (Thomas et al., 2020). This loss of circulating persistent antibodies, in the absence of natural boosting, suggests that HIV infection could result in some loss of long-lived plasma cells in a subset of PWH due to a perturbation of their survival niche in the bone marrow.

### 3.2 HPV vaccine safety, immunogenicity, and efficacy in PWH

There is a growing literature on the performance of HPV vaccination in PWH. Studies show that HPV vaccines have the same safety profile in PWH as in the general population (Staadegaard et al., 2022). Further, vaccination has no impact on CD4^+^ T cell count, HIV viral load, and HIV clinical stage (Denny et al., 2013, Levin et al., 2010, Palefsky et al., 2021b). PWH mount a robust immune response, with some studies showing a correlation with CD4^+^ level and lower immunogenicity in people not on ART (Staadegaard et al., 2022). Peak antibody titers tend to be lower in PWH, but the titers are usually within one log of those seen in the general population and are well above those induced by natural infection (Kojic et al., 2014, Mugo et al., 2018, Palefsky et al., 2021b). Similar to the general population, titers peak one month after the third dose and plateau between 24 and 48 months post vaccination (Mugo et al., 2021). In a study of Gardasil® including 310 perinatally infected children and 148 perinatally HIV-exposed but HIV-uninfected children (PHEU), seropositivity rates were significantly higher among vaccine recipients regardless of the number of doses received as compared to unvaccinated participants. However, antibody titers were lower among CWH than those of PHEU. Surprisingly, antibody titers did not differ considerably between 1-, 2-, and 3-dose recipients for either CWH or PHEU (Moscicki et al., 2019). In a cohort study of adolescents with HIV aged 9 to 15 years with immune reconstitution, antibody responses following a 2-dose regimen were similar to those of subjects aged 15 to 24 years and adolescents without immune reconstitution who received a 3-dose schedule (Rungmaitree et al., 2022). Administration of a fourth vaccine dose to children and adult men who have sex with men (MSM) with HIV elicits a marked rise in antibodies, characteristic of an anamnestic response (Ellsworth et al., 2018, Levin et al., 2010, Weinberg et al., 2018).

Vaccine efficacy data using endpoints of vaccine-type HPV persistent infection and high-grade squamous intraepithelial lesions are very limited among PWH. In 279 WWH followed for a median of 2 years, the rate of vaccine failures (persistent infections and genital warts) was low (1.2/100 person-years) and appeared higher than published data for an HIV-negative vaccinated cohort (0.1/100 person-years). However, the rate of persistent infections in WWH was lower in vaccinated than unvaccinated historical controls (McClymont et al., 2019). Evidence of vaccine efficacy was found among MSM with HIV aged 16 to 26 years with no evidence of previous exposure to HPV vaccine-types compared with those who showed evidence of prior vaccine-type exposure at the time of vaccine initiation (Palefsky et al., 2021a). In contrast, the vaccine offered little to no protection against HPV among MSM with HIV older than 26 years, a population with a high level of prior exposure to HPV vaccine-types (Hidalgo-Tenorio et al., 2021, Wilkin et al., 2018). This is an important issue among PWH because this population has a higher risk of HPV-related cancer than the general population (Stelzle et al., 2021).

These data highlight the importance of vaccinating PWH as early as possible to minimize the likelihood of prior HPV exposure, ideally before initiation of sexual activity. Appendix A presents a summary of results of completed trials of HPV vaccination among PWH.

### 3.3 Impact of HIV acquisition on existing HPV vaccine-induced protection

The effect of HIV on immune response mounted following HPV vaccination may depend on the degree to which HIV is controlled as measured by nadir CD4^+^, current CD4^+^ counts, and possibly HIV viral load. Further, rapid control of HIV infection by early and consistent ART improved preservation of immune function (Kazer et al., 2020). Other factors that might play a role are the number of vaccine doses received and serum titers achieved prior to HIV acquisition. Since, to our knowledge, no data assessing those factors are available, these possibilities need to be evaluated in clinical research studies, as does the impact of any HIV-associated perturbation of immune response on efficacy; however, the design and implementation of such studies may be challenging.

### 3.4 Impact of HIV acquisition on existing vaccine-induced protection: Data from non-HPV vaccines

Given the dearth of data available to quantitate the impact of HIV acquisition on existing HPV vaccine immune response and protection, data from other vaccines, such as hepatitis and smallpox, were reviewed.

#### 3.4.1 Hepatitis vaccines

Hepatitis B (HBV) or hepatitis A (HAV) vaccination programs, in place for decades in high-income settings, could be useful models to investigate how vaccine-induced immunity during childhood or adolescence is affected by HIV infection later in life. However, few data are available on this matter in comparison to what has been generated among those vaccinated after HIV infection. Investigating new clinical hepatitis cases in PWH vaccinated against HBV or HAV during childhood or adolescence compared to a healthy control group is hampered by many confounders, such as shared modes of transmission and increased severity of liver disease in case of HIV co-infection (Lin et al., 2017, Thio et al., 2002). One study in Taiwan, looking at impact of HIV infection in HBV-vaccinated and non-vaccinated cohorts, reports similar declines in hepatitis B core antibodies and hepatitis B surface antigens in persons born in the era of nationwide HBV vaccination, regardless of their HIV status (Sun et al., 2009).

#### 3.4.2 HIV-associated loss of immunity: The example of smallpox vaccination

To understand the impact on protection induced by childhood vaccination by subsequent HIV acquisition, a study was performed among 50 pairs of WWH and HIV-negative women who received smallpox vaccine during childhood. All were born prior to 1971 and were vaccinia virus-seropositive at baseline. Memory T cells and antibody responses were evaluated after subsequent HIV infection, with CD4^+^ T cell nadir < 200/μL that improved to > 350/μL after ART (Thomas et al., 2020). To determine if antigen-specific memory T cells from childhood smallpox vaccination were intact, T cell responses were measured after stimulation with vaccinia virus. A near-complete loss in vaccinia virus-specific CD4^+^ T cell memory was identified among WWH (p = 0.04), whereas antiviral CD8^+^ T cell memory remained intact (p > 0.99).

Longitudinal analyses of vaccinia virus-specific antibody responses were used to measure serological memory over the course of 10 to 21 years. Similar to prior longitudinal studies (Amanna et al., 2007), vaccinia virus-specific antibodies were maintained indefinitely among HIV-negative women (half-life infinity). However, vaccinia virus-specific antibodies significantly declined among WWH (half-life 39 years, p = 0.001). This difference was skewed by a subpopulation (10/50 = 20%) of WWH with antibody half-life estimates of only 2.6 to 8.5 years. These studies indicate that most WWH with CD4^+^ nadir < 200/μL that reconstitute to > 350/μL by ART will lose antigen-specific CD4^+^ T cell memory from prior childhood vaccination, and one-fifth will develop an uncharacteristically rapid loss of vaccine-induced antibodies.

In summary, lessons from vaccination with non-HPV vaccines prior to HIV infection provide limited information for HPV vaccination. Some PWH might lose detectable antibodies over time in the absence of boosting, suggesting that HIV infection could impact long-lived plasma cells. Caution is warranted when extrapolating immunogenicity data to specific risk groups. Although immunological markers are useful as surrogates or correlates of protection against disease, they are not absolute, since those could be proxy for other unestablished vaccine-induced immune responses that may be mediators or contribute to protection. This is illustrated by two randomized trials assessing an inactivated influenza vaccine in PWH with demonstration of high vaccine efficacy despite attenuated vaccine-induced immune responses (Madhi et al., 2011, Madhi et al., 2014). Thus, immunological data need to be complemented with effectiveness studies in specific risk groups.

## 4 Optimizing approaches to HPV vaccine implementation in settings of high HIV prevalence

HPV vaccination is a priority in regions with high prevalence of HIV and cervical cancer, with approximately 6% of all new cervical cancer cases diagnosed globally in 2018 (33,000 new cases) in WWH (Stelzle et al., 2021). Yet the population of WWH, who would benefit most from vaccination, has reduced access to HPV vaccination.

### 4.1 HPV vaccination strategies in a setting of moderate HIV prevalence

To inform programmatic decisions, modeling studies can be used to estimate the impact of vaccination strategies. Using Kenya, a setting of moderate HIV prevalence, as a case study (Liu et al., 2022), a modeling analysis of health impact of several high-coverage nonavalent HPV vaccination strategies on cervical cancer incidence showed that compared to no vaccination, single-age vaccination is projected to reduce cancer incidence by 68% over 50 years in Kenya. In contrast, multi-age cohort vaccination reduces cancer incidence by 75%. Moderate (50%) and high coverage (80%) catch-up vaccination of women aged 15 to 24 or 15 to 44 years reduce cervical cancer incidence by 80 to 84%. These findings suggest that strategies increasing HPV vaccination coverage can be impactful in preventing cervical cancer in settings with moderate HIV burden and where screening and treatment are not readily available. Notably, providing catch-up vaccination to young women and middle-aged women can lead to substantial and rapid reductions of cervical cancer incidence and cases relative to vaccination of 10 to 14-year-olds only.

Even though vaccinating a population likely to have been exposed to HPV might decrease the impact of vaccination, the number of HPV-dysplastic lesions prevented in countries with catch-up vaccination and high coverage, are doubled compared to countries without catch-up vaccination. This is attributed to both direct and indirect herd effects of vaccination (Drolet et al., 2019). Formal cost-effectiveness analyses are needed to evaluate the potential health gains compared to change in costs for vaccination programs that include catch-up vaccination.

### 4.2 Considerations in HPV vaccine delivery strategies by age and risk of HIV acquisition

HPV vaccination programs targeting adolescent girls in high HIV prevalence settings will include, by default, those who will go on to acquire HIV infection as well as those already infected perinatally but undiagnosed. In high HIV prevalence populations, HIV incidence increases rapidly between 15 and 20 years of age (Risher et al., 2021); thus, vaccination of single-age cohorts of 9 to 14-year-olds will likely occur prior to HIV acquisition. Programs should consider how best to reach adolescent girls and women at risk for HIV and HPV to achieve high population coverage prior to exposure to both viruses. In 2021, 91% of PWH above 15 years knew their status (Joint United Nations Programme on HIV/AIDS, 2022); therefore, integration of HPV vaccine services with the HIV clinic platform in high HIV prevalence populations could be an additional approach to promote access to HPV vaccination for either a primary vaccination series or additional vaccine doses following one or two doses received in childhood or adolescence.

Those at risk for HIV and HPV can be reached through school-based vaccination programs, campaign-based vaccination and integration with other routine services like family planning, maternal child health clinics, and vaccination clinics. These service delivery points provide an avenue where there is a ready audience of adolescent girls and young women at risk for HIV and/or HPV infection.

As per WHO recommendations, PWH 9 years of age and older should ideally receive three HPV vaccine doses until further evidence is available for reduced schedules in that population. Considering a 20% HPV vaccination coverage in SSA for a full vaccine schedule (Bruni et al., 2021b), HIV infected children, in practice, often receive one or two doses of HPV vaccine as part of the National vaccine schedule. Should scientific evidence show waning protection among adolescent girls and young women who acquire HIV infection after vaccination with one or two HPV vaccine doses, consideration should be given to providing booster doses to PWH. To reach populations with greatest burden of cervical cancer and attain high coverage, the design of vaccination programs should be people-centered to meet populations at their point of need and in avenues that promote vaccine uptake.

## 5 Knowledge gaps and outstanding research questions

Outstanding research questions exist in the fields of epidemiology, immunology, and vaccinology in PWH, as well as the intersection of HPV vaccination implementation and HIV infection. Notably, the key question posed to the experts—will immunocompetent girls at time of HPV vaccination who acquire HIV thereafter have similar HPV vaccine protection as those who are HIV-negative—remains unanswered. Although a direct clinical investigation of this question would be large and challenging to conduct, several ongoing field studies may have the framework to monitor cohorts of immunocompetent girls at high risk for HIV acquisition. Examples of such studies include a study of unvaccinated girls with and without HIV in Botswana (McClung et al., 2022) who will serve as a baseline against which to compare vaccine effectiveness in subsequent cohorts of vaccinated girls, by HIV status; the STAR study (Sheth et al., 2021) in the United States that recruited 2,000 reproductive-age women with and without HIV, many of whom will have been HPV-vaccinated prior to HIV acquisition; and a study in Rwanda of girls, who received HPV vaccination as adolescents and later acquired HIV (personal communication). These studies are complicated by difficulties in confirming individual HPV-vaccination status (including number of doses received) and HIV status. Yet, they raise the possibility of quantitating vaccine effectiveness and durability of protection and could thus be reassuring. Alternatively, if reduced vaccine protection is observed in HPV vaccinees post HIV-acquisition, explorations of alternative approaches to offset compromised protection, such as additional vaccine dose, alternative schedules, or improved vaccine formulation, may be required.

Beyond studying individual-level protection, vaccine impact studies that nest cross-sectional HPV prevalence surveys in PWH and immunocompetent individuals may address relevant questions. Notably, the HOPE trial in South Africa (Machalek et al., 2022) has the possibility to investigate HPV protection, including the protection elicited by a single dose, in WWH and women who acquire HIV after HPV vaccination.

Although we are ultimately interested in protection against HPV infection, immunologic studies could be instructive to understand if the HPV-vaccine-induced antibodies produced prior to HIV acquisition are quantitatively and qualitatively similar after HIV infection. For this work, laboratory studies should investigate antibody binding, neutralization, and other functionalities. Inferences can be made based on HPV vaccination among WWH by comparing antibody profiles to an appropriately matched cohort of immunocompetent women. If minimal differences are observed, this strengthens the hypothesis that HPV-vaccine-induced antibodies will remain sufficiently robust even after HIV acquisition. Confirming that circulating antibodies reflect antibodies at local and mucosal anatomic sites in WWH, assessing the role of the vaginal microbiome, and addressing how disruption of the epithelium by HIV impacts susceptibility to infection and the antibody-mediated protection of HPV vaccines, are important questions largely unexplored.

Among WWH, there is a robust pipeline of studies initiated in recent years that attempts to address open questions on dosing as well as vaccine effectiveness in different settings. Although the recently updated WHO guidance for HPV dosing among WWH is to receive at least 2 doses, with 3 doses being preferred, several ongoing and new HPV vaccine trials using immunologic and virologic endpoints will generate evidence in WWH on reduced-dose HPV schedules within the next 5 years. Studies in WWH comparing antibody responses of 2 versus 3 doses are ongoing in Africa (Eswatini, Rwanda), Latin America (Brazil, Peru), and Europe (Belgium). There is also an HPV vaccine effectiveness study among WWH comparing 1 to 3 doses of the bivalent, quadrivalent, or nonavalent HPV vaccines in SAA (see Table 1 for full list of trials). These studies are modest in size but will provide additional guidance to policymakers for dosing and may assist in setting expectations around residual burden of HPV-related disease among vaccinated WWH. Similar studies of new HPV vaccines will be important to inform countries making implementation decisions, including tradeoffs if the cost and availability of higher valency vaccines make access difficult.

**Table 1:** Ongoing HPV vaccination trials among girls and women with HIV registered in the US. National Library of Medicine database and WHO clinical trials databasea.

Trial acronym *Location* (Clinical trial number)	Description	HIV viral load or CD4 count at enrolment	Currently receiving ART	Vaccine product	Population and age (years)	Sample size	Primary aim(s)	Estimated study primary completion
Recruiting								
9-VPH-MVIH *Spain* (NCT04270773)	Phase IV, open-label, single-arm trial of the 9vHPV vaccine in HIV^+^ women not infected simultaneously by HPV16/HPV18 in vagina and/or anus.	≥200 cells/µL	ND	9vHPV 3-doses (0, 2, 6 M)	WWH 18+	158	Immunogenicity and safety at M30	2022
-- *Eswatini* (NCT04982614)	Phase IV, multi-site, open-label non-inferiority trial to assess immunogenicity of 2 doses of the 9vHPV vaccine among boys and girls (9–14 years) and young women (15–26 years) with HIV vs. 3 doses of 9vHPV vaccine among HIV-negative young women (15–26 years).	ND	PWH: use 6 + months at enrollment	9vHPV 2 doses (0, 6 to 12 M) 9vHPV 3 doses (0, 2, 6 M)	Boys & girls (9–14) with HIV & WWH 15–26 & HIV^-^ women 15–26	1400	Immunogenicity (HPV GMTs and seroconversion) at M7	2023
COVENANT *Kenya, Malawi, South Africa, Uganda, Zimbabwe* (NCT03284866)	Phase III, randomized, placebo-controlled trial of HPV vaccination to reduce cervical HSIL among HIV-infected women participating in an HPV test-and-treat program.	ND	6 + months prior to randomization	9vHPV 3 doses vs. placebo (0, 1, 6.5 M)	HIV^+^ women 25+	536	Efficacy (occurrence of HSIL or cervical cancer) 12 M post randomization	2023
OPTIMO *Brazil, Peru* (NCT04265950)	Phase IV, multicenter, randomized, open-label trial in children and adolescents with HIV to establish the optimal number of HPV vaccine doses.	ND	>6 months prior to study enrolment	9vHPV3 (0, 2, 6 M) vs. 2 doses (0, 6 M) All arms receive a booster at 30 M	Boys & girls with HIV & HIV^-^ participants 9–13	100	Immunogenicity (HPV16 GMTs) at M24 in PWH	2025

Active, not recruiting								
Papillon *Belgium* (NCT03391921)	Phase IV, randomized, parallel assignment, open-label, trial on immunogenicity, induction of cellular immune responses and safety of 9vHPV vaccine in HIV^+^ women.	Undetectable for 6 + months (HIV RNA < 400 cp/ml)	ND	9vHPV2 (0, 6 M) & 3rd dose 18–48 M if antibody levels are insufficient) vs. 3-doses (0, 2, 6 M)	HIV + women 15–40	170	Immunogenicity at M7	2023
-- *Kenya*^b^ (NCT05435209)	Phase IV, single group assignment, open-label longitudinal cohort study to assess effectiveness and B cell memory response to the 4vHPV vaccine 9 years post-vaccination among HIV-infected adolescents who received the HPV vaccine at age 9–14 years.	ND	ND	4vHPV 3-doses (0, 2, 6 M)	4vHPV vaccinated HIV^+^ individuals 16–25	174	Effectiveness (persistent genital HPV infection) & immunogenicity(long-term antibody response to the 4vHPV types) 9 years post vaccination	2023

Other (cohort)								
-- *Kenya*^b^ (NCT04920526)	HIV-infected adolescents who received 3 doses of the 4vHPV vaccine in the 4vHPV vaccine safety and immunogenicity study (MISP 38406) at age 9–14 years. [Not yet recruiting]	ND	ND	4vHPV 3-doses	4vHPV vaccinated HIV^+^ boys & girls 16–24	135	Effectiveness & Immunogenicity (changes in persistent genital HPV infection, 4vHPV GMTs) 9 years post vaccinationB cell memory & T cells (after booster vaccine) at 12 M	2022
SHiP *England* (NCT04587050)	Prevalence of cervical cytology abnormalities, high-risk HPV status, and HPV antibody titers in adult women with perinatally-acquired HIV infection. HPV vaccinated and unvaccinated women are eligible to participate. [Recruiting]				Women with perinatally-acquired HIV 18 years or older who are sexually active or not.		Prevalence of abnormal cervical cytology at 12 M	2023
-- *Rwanda* (NCT05247853)	Effectiveness of prophylactic HPV vaccine in reducing cervical, anal, and/or oral prevalent and 6-month persistent infections in WWH and HIV^-^ women vaccinated with 2 or 3 doses of 4vHPV at age 12, and in unvaccinated WWH (number of doses received is dictated by introduction of HPV vaccination in 2011). [Recruiting]	ND	ND	4vHPV 0 vs. 2 or 3 doses	HPV-vaccinated & unvaccinated WWH & HPV-vaccinated HIV^-^ women 18–26	2271	Effectiveness (6-month persistent cervicovaginal, anal, and oral infections)	2025
				4vHPV 3 vs. 2 doses			Immunogenicity (changes in antibody response) at 6 M	

Abbreviations: 4vHPV, Quadrivalent HPV vaccine (GARDASIL®, Merck Sharp & Dohme Corp.); 9vHPV, Nonavalent HPV vaccine (GARDASIL®9, Merck Sharp & Dohme Corp.); ART, Antiretroviral therapy; GMTs, Geometric mean antibody titers; HIV, human immunodeficiency virus; HPV, human papillomavirus; HSIL, high-grade squamous intraepithelial lesions; M, month; ND, no data provided; PWH, people with HIV; WHO, World Health Organization; WWH, women with HIV.

a Search strategy: Clinical trials were identified using “HPV vaccine” AND “HIV” in the US National Library of Medicine database of privately-and publicly funded clinical studies conducted around the world (ClinicalTrials.gov) and the World Health Organization International Clinical Trials Registry Platform (https://trialsearch.who.int/). The search was limited to trials that are recruiting or are active but not recruiting, and identified 11 studies. We excluded 2 studies: men only (NCT04255849) and therapeutic vaccine (NCT03603808). We also searched the “not yet recruiting category” (n = 6 studies), excluded 1 behavioral study (NCT05065840), and contacted the principal investigators of 5 studies to gather information on whether recruiting had started.

b Same cohort of vaccinated children and adolescents in Kenya.

## 6 Conclusion

A workshop was convened to discuss relevant data to inform two questions: what is the performance of HPV vaccination in WWH, and does HIV acquisition after HPV vaccination of young girls negatively impact vaccine-mediated protection? The experts also discussed the number of HPV vaccine doses required in these two scenarios. Trends in improved perinatal ART use and decreased HIV prevalence among adolescents will likely lead to a higher proportion of women vaccinated when immunocompetent, making the second question more relevant. With no direct data on this topic and limited information from non-HPV vaccines, this question necessitates further research. Studies of HPV vaccination among WWH are more common and, although recommendations for use of a multi-dose HPV regimen are in place, we anticipate enhanced data on vaccine effectiveness and refined best practices over the next 2 to 3 years. Although modeling suggests that broader use of HPV vaccines among a wider age range of girls and women can be highly impactful to protect WWH, implementation research to understand optimal HPV delivery approaches for at-risk populations and WWH is urgently needed. The disproportionate impact of cervical cancer among WWH and the many open research questions on use of HPV vaccines highlight the important work ahead to address this difficult problem.

## Funding

Financial support for this work was provided to PATH by the Bill & Melinda Gates Foundation, Seattle, WA (grant ID INV-008475).

## CRediT authorship contribution statement

**Anne E. Schuind:** Conceptualization, Writing – original draft, Writing – review & editing. **Helen Rees:** Writing – review & editing. **John Schiller:** Writing – review & editing. **Nelly Mugo:** Writing – original draft, Writing – review & editing. **Peter Dull:** Conceptualization, Writing – review & editing. **Ruanne Barnabas:** Writing – review & editing. **Gary M. Clifford:** Writing – original draft, Writing – review & editing. **Gui Liu:** Writing – original draft, Writing – review & editing. **Shabir A. Madhi:** Writing – original draft, Writing – review & editing. **Rebecca B. Morse:** Writing – original draft, Writing – review & editing. **Anna-Barbara Moscicki:** Writing – review & editing. **Joel M. Palefsky:** Writing – original draft, Writing – review & editing. **Stanley Plotkin:** Writing – review & editing. **Mónica S. Sierra:** Writing – original draft, Writing – review & editing. **Mark K. Slifka:** Writing – original draft, Writing – review & editing. **Alex Vorsters:** Writing – original draft, Writing – review & editing. **Aimée R. Kreimer:** Conceptualization, Writing – original draft, Writing – review & editing. **Arnaud M. Didierlaurent:** Writing – original draft, Writing – review & editing.

## Declaration of Competing Interest

The authors declare the following financial interests/personal relationships which may be considered as potential competing interests: AMD reports participation in advisory boards for Sanofi, Bioaster, Speranza and ACM Biolabs, and research grants from GlaxoSmithKline, Moderna, and Roche, outside the submitted work. ABM reports personal fees from Merck & Co. global advisory board honorarium, outside the submitted work. GL is an employee of Merck & Co. NM is a recipient of an Investigator Initiated Grant evaluating ‘Immunogenicity of HPV vaccine among HIV infected adolescents’, outside the submitted work. JMP reports grants and personal fees from Merck & Co., from Vir Biotechnologies, from Antiva Biosciences, from Roche Diagnostics, and other from Virion Therapeutics, outside the submitted work. JS reports participation to DSMB for NCT04508309 and the India/IARC Gardasil trial. SAM reports grants from Bill & Melinda Gates Foundation, Pfizer, GlaxoSmithKline, Minervax and Sanofi, outside the submitted work. SP reports personal fees from Merck & Co. global advisory board honorarium, and personal fees from Sanofi, Inovio, Merck & Co., Janssen, Moderna, Valneva, Codagenix, Pfizer, VaxArt, Meissa, Vaxinnity, Rational, and AstraZeneca, outside the submitted work. RB reports grants from National Institutes of Health and Bill & Melinda Gates Foundation and non-financial support from Regeneron Pharmaceutical, outside the submitted work. All other authors declare that they have no known competing financial interests or personal relationships that could have appeared to influence the work reported in this paper.

## Data Availability

No data was used for the research described in the article.
