# Supplementary material for: State-of-the-Science of human papillomavirus vaccination in women with human immunodeficiency Virus: Summary of a scientific workshop
Source: Prev Med Rep. 2023 Jul 19;35:102331. doi: 10.1016/j.pmedr.2023.102331 (PMC10413150; doi:10.1016/j.pmedr.2023.102331)
Supplement: Supplementary data 1 [file mmc1.docx]

**Appendix A**

State-of-the-Science of Human Papillomavirus Vaccination in Women with Human Immunodeficiency Virus: Summary of a Scientific Workshop.

Authors: Schuind A, Rees H, Schiller J, Mugo N, et al.

**Literature review**

Aim: To obtain summary estimates of vaccine efficacy, immunogenicity, and other endpoints (excluding safety) of HPV vaccination in people with HIV (PWH) to inform HPV vaccination in women and girls with HIV.

**Search strategy**

Clinical trials were identified using “HPV vaccine” AND “HIV” in the US National Library of Medicine database of privately and publicly funded clinical studies conducted around the world (ClinicalTrials.gov) and the World Health Organization International Clinical Trials Registry Platform (<https://trialsearch.who.int/>). The search was limited to trials that were completed, terminated, or suspended. We excluded studies focusing only on men (NCT00513526, NCT02503111, NCT02087384, NCT01923116, NCT01209325).

We also hand-searched PubMed to identify trial results using the registered trial ID or the protocol number and allowed multiple entrances for the same study. We identified 22 studies. We excluded 7 studies: HIV-negative only (NCT01489527), therapeutic vaccination in treated HIV disease (NCT03606213), no HPV vaccine/prevalence/behavioral study (NCT01311752, NCT01788852, NCT04401670, NCT00840905), and no results available (NCT00798265).

We included 16 unique studies (23 published reports). We also checked our review against the recent systematic review and meta-analysis by Staadegaard et al. (Staadegaard et al., 2022). We included 3 reports (Firnhaber et al., 2021; Kojic et al., 2014; Palefsky et al., 2014) not incorporated in Staadegaard’s review.

**Supplementary Table 1. Summary results of published efficacy, immunogenicity, and other endpoints from completed HPV vaccine trials among HIV-infected subjects (trials in men only were excluded)**

| **Location**  **Clinical Trial No.**  **(Reference)** | **Description** | **Vaccine product and no. doses**  **(schedule)** | **Population and age (years)** | **Sample size** | **Timing of measure**  **(months)** | **Results** | |
| --- | --- | --- | --- | --- | --- | --- | --- |
| **Efficacy endpoint** | | | | | | | |
| **South Africa**  NCT01928225  (Firnhaber et al., 2021) | Phase II randomized, blinded, placebo-controlled trial of pre-treatment HPV vaccination on outcomes to LEEP treatment of cervical HSIL in HIV-infected women (QHPV-RTC) | 4vHPV  3 doses  (0, 2, 6M) | WWH  39.2  (median)  (34.9-45.5) | 174 | 6 | Recurrence of cervical HSIL (histology or cytology) | Risk ratio (95% CI)  1.2 (0.87; 1.6) |
|  |  |  |  |  |  | Recurrence of histologic HSIL (CIN2 or CIN3) | 1.04 (0.67; 1.6) |
|  |  |  |  |  |  | Recurrence of CIN3 | 0.82 (0.36; 1.9) |
| **United States, Puerto Rico, Brazil**  NCT01461096  (Wilkin et al., 2018) | Phase III, double-blind, randomized, placebo, controlled trial to evaluate the effectiveness of the 4vHPV vaccine at reducing the incidence of 4M persistent anal/oral HPV infections in HIV-infected MSM and HIV-infected women. Ended early due to futility.  (ACTG A5298) | 4vHPV  3 doses  (0, 2, 6M) | PWH  ≥27 years  [30% of men & 50% of women with biopsy-confirmed anal HSIL] | 575  (82% men; 18% women)  286 vs. 283  276 vs. 277 | 48 | Persistent anal infection  mITT  Per protocol analysis | VE (95% CI):  21% (−61%; 61%)  31% (−82%; 74%) |
|  |  |  |  | 288 vs. 286  278 vs. 280 |  | Persistent oral infection  mITT-persistent infection only  Per protocol analysis | 88% (2%; 98%)  66% (–70%; 96%) |
|  |  |  |  | 288 vs. 286 |  | Improvement of anal HSIL (biopsy outcomes)  Full ITT | 0% (–44%; 31%) |
|  |  |  |  | 231 vs. 229  199 vs. 198  130 vs. 132 | 12  26  39 | Abnormal anal cytology  Abnormal anal cytology  Abnormal anal cytology | 0% (–19%; 16%)  9% (–10%; 25%)  17% (–6%; 35%) |

| **Location**  **Clinical trial No.**  **(Reference)** | **Description** | | **Vaccine product**  **No. Doses**  **(schedule)** | | **Population**  **Age (years)** | | **Sample size** | **Timing of measure**  **(months)** | **Results** | | | |
| --- | --- | --- | --- | --- | --- | --- | --- | --- | --- | --- | --- | --- |
| **Immunogenicity endpoint** | | | | | | | | | **Assay** | **HPV type** | **GMT (95% CI)** | **Seroconversion**  **% (95% CI)** |
| **Kenya**  NCT04711265  (Mugo et al., 2018) | Single arm trial to assess sustained immunogenicity of the 4vHPV vaccine among HIV-infected girls and boys. (MISP 38406) | 4vHPV  3 doses  (0, 2, 6M) | | HIV infected boys and girls  9-14 | | 179 | | 7 | cLIA  (mMU/mL) | HPV16 | 2322 (1912; 2820) | 98.3 |
|  |  |  |  |  |  |  | |  |  | HPV18 | 364 (289; 458) | 93.3 |
|  |  |  |  |  |  | 178 | | 12 |  | HPV16 | 657 (529; 817) | 97.8 |
|  |  |  |  |  |  |  |  |  |  | HPV18 | 89 (71; 112) | 72.5 |
| NCT01998178,  NCT01446718  (Mugo et al., 2021)  [(MISP)IISP 51802] | HIV+ vs. HIV- (historical control) |  | |  | | 176 | | 24 |  | HPV16 | 243 (183; 322)  944 (804; 1108) | 96 |
|  |  |  | |  | |  |  |  |  | HPV18 | 39 (29; 52)  138 (115; 165) | 82 |
|  | HIV+ vs. HIV- (historical control) |  | |  | | 174 | | 36 |  | HPV16 | 170 (126; 230)  642 (562; 733) | 93 |
|  |  |  | |  | |  |  |  |  | HPV18 | 29 (21; 39)  87 (75; 101) | 78 |
|  | HIV+ |  | |  | | 164 | | 48 |  | HPV16 | 137 (100; 187) | 90 |
|  |  |  | |  | |  |  |  |  | HPV18 | 23 (17; 31) | 77 |
| **Belgium**  NCT03525210, NCT03482739  (Boey et al., 2021) | Phase III, single-center, open-label study on safety, tolerability and immunogenicity of 9vHPV vaccine among HIV-infected persons (CD4-count ≥200 cells/uL and viral loads below the detection limit) and SOT patients. (Protocol V503-044-IC, EUCTR2017-004322-15-BE) | 9vHPV  3 doses  (0, 1, 6M) | | Men & women  18-45  18-55 | | 271  HIV (n=100)  SOT (n=171) | | 7 | cLIA  (mMU/mL) |  | PPI population |  |
|  |  |  |  |  |  |  |  |  |  |  |  |  |
|  |  |  | | HIV  All SOT | | 63  149 | |  |  | HPV16 | 2589 (2096; 3197)  170 (123; 234) | 100 (94.3; 100)  69.1 (61.0; 76.4) |
|  |  |  | | HIV  All SOT | | 67  143 | |  |  | HPV18 | 613 (497; 757)  84 (72; 98) | 100 (94.6; 100)  51.7 (43.2; 60.2) |

| **Location**  **Clinical trial No.**  **(Reference)** | **Description** | **Vaccine product**  **No. Doses**  **(schedule)** | **Population**  **Age (years)** | **Sample size** | **Timing of measure**  **(months)** | **Results** | | | |
| --- | --- | --- | --- | --- | --- | --- | --- | --- | --- |
| **Immunogenicity endpoint** | | | | | | **Assay** | **HPV type** | **GMT (95% CI)** | **Seroconversion**  **% (95% CI)** |
| **Brazil, Estonia, India, Thailand**  NCT01031069  (Folschweiller et al., 2020) | Phase IV, randomized, blind, controlled, multicentric study to compare the safety and immunogenicity of the 2vHPV and 4vHPV vaccines in HIV+ women.  (Protocol 2013-003429-28, CTRI/2009/091/ 001039) | 2vHPV  3 doses  (0, 1.5, 6M)  vs.  4vHPV  3 doses  (0, 1.5, 6M) | Women  15-25 | WWH (ATP)  80 vs. 83 | 7 | PBNA | HPV16  HPV18 | Non-inferiority  Adjusted GMT ratio:  2.95 (1.92; 4.52)  7.83 (4.84; 12.66) |  |
|  |  | 2vHPV vs. 4vHPV |  | WWH (TVC)  109 vs. 110 | 7 | PBNA | HPV16  HPV18 | Superiority analysis  Adjusted GMT ratio  2.74 (1.83; 4.11)  7.44 (4.79; 11.54) |  |
|  |  | 2vHPV vs. 4vHPV |  | Women w/o HIV (TVC)  105 vs. 102 |  |  | HPV16  HPV18 | 3.05 (1.84; 5.06)  5.38 (3.20; 9.06) |  |
|  | WWH (2vHPV) vs. women w/o HIV (4vHPV) |  |  | ATP  80 vs. 80 | 7 | PBNA | HPV16  HPV18 | Superiority analysis  Adjusted GMT ratio  0.83 (0.57; 1.20)  1.77 (1.20; 2.61) |  |
|  | CD4 T-cell response median frequency in initially seronegative WWH | 2vHPV vs. 4vHPV |  | 14 vs. 10 | 12 |  | HPV16 | Median (IQR)  2155.0 (1225.0; 4614.0)  1715.0 (1106.0; 2511.0) |  |
|  |  |  |  | 11 vs. 13 |  |  | HPV18 | 1715.0 (1070.0; 4063.0)  987.0 (346.0; 1932.0) |  |
|  | Memory B-cell response in initially seronegative WWH | 2vHPV vs. 4vHPV |  | 18 vs. 8 | 12 |  | HPV16 | Median (IQR)  256.5 (45.0; 1003.0)  180.0 (64.5; 476.0) |  |
|  |  |  |  | 16 vs. 10 | 12 |  | HPV18 | 103.0 (1.0; 705.5)  37.5 (1.0; 102.0) |  |

| **Location**  **Clinical trial No.**  **(Reference)** | **Description** | **Vaccine product**  **No. Doses**  **(schedule)** | **Population**  **Age (years)** | **Sample size** | **Timing of measure**  **(months)** | **Results** | | | |
| --- | --- | --- | --- | --- | --- | --- | --- | --- | --- |
| **Immunogenicity endpoint** | | | | | | **Assay** | **HPV type** | **GMT (95% CI)** | **Seroconversion**  **% (95% CI)** |
| **United States, Puerto Rico**  (Levin et al., 2010) | Randomized, double-blinded, placebo-controlled trial of the safety and immunogenicity of the 4vHPV vaccine in HIV-infected children. (IMPAACT P1047, NCT00339040) | 4vHPV  3-doses  vs. placebo | HIV infected children  7-11 | All 4vHPV  (n=96)  vs placebo (n=30) | 7 | cLIA  (mMU/mL) | HPV16  HPV18 | 5231 (4108; 6660)  931 (656; 1321) | 4vHPV vs placebo  100 vs 4  97 vs 0 |
|  | CD4% nadir < 15 and CD4% ≥ 15 at screening |  |  | 4vHPV (n=31) |  |  | HPV16  HPV18 | 5984 (3617; 9900)  1078 (506; 2300) | 100 vs. 0  90 vs. 0 |
|  | CD4% nadir ≥ 15 and CD4%  15 – 24 at screening |  |  | 4vHPV (n=32) |  |  | HPV16  HPV18 | 5129 (3224; 8161)  984 (544; 1779) | 100 vs. 10  100 vs. 0 |
|  | CD4% nadir ≥ 25 and CD4% ≥ 25 at screening |  |  | 4vHPV (n=33) |  |  | HPV16  HPV18 | 4700 (3394; 6508)  759 (455; 1268) | 100 vs. 0  100 vs. 0 |
|  | HIV+  HIV – (historical controls) |  | 9-12 | 65  560 |  |  | HPV16 | 4987 (3685; 6751)  6444 (5840; 7110) |  |
|  | HIV+  HIV – (historical controls) |  | 9-12 | 65  565 |  |  | HPV18 | 845 (547; 1306)  1558 (1416; 1716) |  |
| **United States, Puerto Rico**  (Weinberg et al., 2012) | HPV type-specific antibodies 18M after 3-dose or 4-dose series of 4vHPV vaccine in HIV-infected children. Mucosal and CMI responses to vaccine genotypes and cross-reactive responses to HPV31 (IMPAACT P1047). | 4vHPV  4 dose  (0, 2, 6, 24M)  vs. 3 doses  (0, 2, 6M –initial placebo group) | HIV infected children  7-11 | 126  4 doses (n=96)  3 doses (n=30) | 1  after 3^rd^ dose | cLIA  (mMU/mL) | HPV16  HPV18 |  | 100 vs. 100  97 vs. 100 |
|  |  |  |  |  | 18  after 3^rd^ dose |  | HPV16  HPV18 | Declines from 2.2-fold to 6.3-fold for all genotypes | 99  76 |
|  |  |  |  |  | 0.25  after 4^th^ dose |  | HPV16  HPV18 | Geometric mean fold-rise  6  10 | 100  96 |
| NCT01206556  (Levin et al., 2017) | Duration of HPV type-specific antibody after 4vHPV vaccine in HIV-1 infected children previously enrolled in IMPAACT P1047. (IMPAACT P1085) | 4vHPV  4 doses  (0, 2, 6, 24M) vs. 3 doses  (0, 2, 6M –initial placebo group) | HIV infected Children  7-11 | 97  4 doses (n=58)  3 doses (n=14) | 48-60 | cLIA  (mMU/mL) | HPV16 | 1206 (780; 1863)  310 (83; 1160) | 98  86 |
|  |  |  |  |  |  |  | HPV18 | 108 (64; 183)  42 (15; 117) | 74  64 |

| **Location**  **Clinical trial No.**  **(Reference)** | **Description** | **Vaccine product**  **No. Doses**  **(schedule)** | **Population**  **Age (years)** | **Sample size** | **Timing of measure**  **(months)** | **Results** | | | |  |
| --- | --- | --- | --- | --- | --- | --- | --- | --- | --- | --- |
| **Immunogenicity endpoint** | | | | | | **Assay** | **HPV type** | **GMT (95% CI)** | **Seroconversion**  **% (95% CI)** |  |
| **India**  NCT00667563  (Palefsky et al., 2021) | Phase I, single-arm, open-label pilot study of the safety and immunogenicity of 4vHPV vaccine among HIV-positive women. CTRI/2009/091/ 000298, AMC 054) | 4vHPV  3 doses  (0, 2, 6M) | WWH  19-44 | 150 | 7 | cLIA  (mMU/mL) | HPV16  HPV18 |  | ATP  99  90 |  |
|  |  |  |  |  | 12 |  | HPV16  HPV18 |  | 99  71 |  |
| (Palefsky et al., 2014) | Seronegative and DNA-negative at baseline |  |  |  |  |  |  |  |  |  |
|  | CD4 nadir ≤ 350 on HAART |  |  |  | 7  13 |  | HPV16 | 2271  771 |  |  |
|  | CD4 nadir >350, CD4 350-500, not on HAART |  |  |  | 7  13 |  |  | 2205  484 |  |  |
|  | CD4 nadir >350, CD4 >500, not on HAART |  |  |  | 7  13 |  |  | 3045  730 |  |  |
|  | CD4 nadir ≤ 350 on HAART |  |  |  | 7  13 |  | HPV18 | 183  33 |  |  |
|  | CD4 nadir >350, CD4 350-500, not on HAART |  |  |  | 7  13 |  |  | 198  35 |  |  |
|  | CD4 nadir >350, CD4 >500, not on HAART |  |  |  | 7  13 |  |  | 270  38 |  |  |
| **Canada**  ISRCTN33674451  (Money et al., 2016) | Open-label, multicenter, single-arm study evaluating immunogenicity and safety of 4vHPV vaccine among women and girls with HIV. | 4vHPV  3 doses  (0, 2, 6M) | WWH  15-66 | 310 | 7 |  | HPV16  HPV18 |  | PP  98.1  93.6 |  |
|  |  |  |  |  | 24 |  | HPV16  HPV18 |  | 97.7  67.0 |  |
|  | Suppressed vs. unsuppressed viral load |  |  | PP  84 vs. 28  111 vs. 34 |  | cLIA  (mMU/mL) | HPV16  HPV18 | GMT ratio  1.74 (1.17; 2.59)  3.05 (1.94; 4.80) |  |  |
|  | HIV- (historical control) vs. HIV+ |  | 15–26 | 2573 vs. 18 | 7 |  | HPV16 | 2294 (2185; 2408)  3473 (2168; 5566) |  |  |
|  |  |  |  | 2800 vs. 19 |  |  | HPV18 | 462 (444; 480)  334 (189; 591) |  |  |
|  |  |  |  | 2381 vs. 12 | 24 |  | HPV16 | 460 (441; 481)  319 (167; 610) |  |  |
|  |  |  |  | 2603 vs. 14 |  |  | HPV18 | 52 (49; 55)  16 (9; 27) |  |  |
| **Location**  **Clinical trial No.**  **(Reference)** | **Description** | **Vaccine product**  **No. Doses**  **(schedule)** | **Population**  **Age (years)** | **Sample size** | **Timing of measure**  **(months)** | **Results** | | | |  |
| **Immunogenicity endpoint** | | | | | | **Assay** | **HPV type** | **GMT (95% CI)** | **Seroconversion**  **% (95% CI)** |  |
| **Canada**  ISRCTN33674451  (Brophy et al., 2018) | Sub-study of open-label, multicenter Canadian study evaluating the immunogenicity and safety of 4vHPV vaccine among girls with HIV. | 4vHPV  3 doses  (0, 2, 6M) | Girls with HIV  9-13 | 35 | 7 | cLIA  (mMU/mL) | HPV16  HPV18 |  | 100  100 |  |
|  |  |  |  |  | 24 |  | HPV16  HPV18 |  | 100  72.0 |  |
|  | HIV+ vs. HIV- (historical control) |  |  | 32 vs. 251 | 7 | cLIA  (mMU/mL) | HPV16 | Age adjusted GMT ratio  0.52 (0.33; 0.82) |  |  |
|  | HIV+ vs. HIV- (historical control) |  |  | 31 vs. 252 |  |  | HPV18 | 0.31 (0.20; 0.49) |  |  |
|  | HIV+ vs. HIV- (historical control) |  |  | 26 vs. 186 | 24 | cLIA  (mMU/mL) | HPV16 | 0.36 (0.24; 0.55) | 100 vs. 100 |  |
|  | HIV+ vs. HIV- (historical control) |  |  | 25 vs. 187 |  |  | HPV18 | 0.22 (0.12; 0.40) | 72.0 vs. 93.6 |  |
| **Canada**  ISRCTN33674451  (Moscicki et al., 2019) | Cohort study to compare antibody titers against 4vHPV types and rate of abnormal cytology between  perinatally HIV-infected (PHIV) and perinatally HIV-exposed, uninfected (PHEU) youth part of the Adolescent Master Protocol for Participants 18 Years of Age and Older (AMP Up; NCT02119702). | 4vHPV recipients (1, 2, or 3 doses) | Children  7-16 | Vaccinated population:  278 PHIV vs. 115 PHEU  [1-dose : 154 vs 91 ; 2-doses: 34 vs 13; 3-doses: 90 vs 11] | Median 35  (IQR: Q1, Q3  22, 49) | cLIA  (mMU/mL) | HPV16  HPV18 |  | 90% vs. 99%  62% vs. 87% | |
|  | PHIV Youth | 1 dose (ref.)  2 doses  ≥3 doses |  |  |  |  | HPV16 | Antibody titers change  1.55 (−1.30; 3.15)  −1.06 (−2.01; 1.79) |  | |
|  |  | 1-dose (ref.)  2-doses  ≥3 doses |  |  |  |  | HPV18 | 1.52 (−1.21; 2.80)  −1.24 (−2.16; 1.41) |  | |
|  | CD4% ≥ 25% at first vaccine  dose | At least one vaccine dose |  |  |  |  | HPV16  HPV18 | 1.85 (1.04; 3.29)  1.43 (−1.16; 2.35) |  | |
|  | HIV RNA (copies/mL) <400 (ref.)  400 to <1000  ≥1000 | At least one vaccine dose |  |  |  |  | HPV16 | −3.70 (−8.55; −1.60)  −4.09 (−7.12; −2.34) |  | |
|  | 400 to <1000  ≥1000 |  |  |  |  |  | HPV18 | −2.23 (−4.61; −1.07)  −2.91 (−4.72; −1.80) |  | |
|  | On 3+ months continuous cART at first vaccine dose | At least one vaccine dose |  |  |  |  | HPV16  HPV18 | −1.07 (−1.95; 1.71)  1.60 (−1.05; 2.71) |  | |

| **Location**  **Clinical trial No.**  **(Reference)** | **Description** | **Vaccine product**  **No. Doses**  **(schedule)** | **Population**  **Age (years)** | **Sample size** | **Timing of measure**  **(months)** | **Results** | | | |
| --- | --- | --- | --- | --- | --- | --- | --- | --- | --- |
| **Immunogenicity endpoint** | | | | | | **Assay** | **HPV type** | **GMT (95% CI)** | **Seroconversion**  **% (95% CI)** |
| **Denmark**  NCT01386164  (Toft et al., 2014) | Phase IV, randomized, double-blind trial to compare the immunogenicity and reactogenicity of 2vHPV and 4vHPV in HIV-infected adult men and women. | 2vHPV vs. 4vHPV | HIV+ women  ≥18 | 15 vs. 15 | 7  12 | cLIA  (mMU/mL) | HPV16 | GMT ratio  2.98 (0.76; 11.6)  3.90 (1.05; 14.6) |  |
|  |  |  |  |  | 7  12 |  | HPV18 | 3.93 (1.02; 15.2)  6.02 (1.05; 14.6) |  |
|  |  | 2vHPV vs. 4vHPV | HIV+ men  ≥18 | 30 vs. 31 | 7  12 | cLIA  (mMU/mL) | HPV16 | No difference in GMTs 2vHPV vs 4vHPV |  |
|  |  |  |  |  | 7  12 |  | HPV18 | 4.54 (2.15; 9.59)  3.55 (1.48; 8.53) |  |
| **United States, Puerto Rico**  NCT00710593  (Kahn et al., 2013) | Phase II, open-label, multicenter trial to evaluate the immunogenicity and safety of 4vHPV vaccine in HIV-infected young women. | 4vHPV  3 doses  (0, 2, 6M) | Women  16-23 | 99  (69 not ART; 30 ART) |  |  |  | Mean GMT (PP) |  |
|  | non-ART group |  |  | 42 | 7 | cLIA  (mMU/mL) | HPV16 | 2393 (1252; 3534) | 96.4 (81.7; 99.9) |
|  | ART group |  |  |  |  |  |  | 5046 (2338; 7755) | 100 (75.3; 100.0) |
|  | Historical comparison (negative for all 4vHPV types) |  |  | 267 |  |  |  | 3892 (3324; 4558) | 100 (--) |
|  | non-ART group |  |  | 57 |  |  | HPV18 | 463 (247; 679) | 92.3 (79.1; 98.4) |
|  | ART group |  |  |  |  |  |  | 979 (302; 1655) | 100 (79.4; 100.0) |
|  | Historical comparison (negative for all 4vHPV types) |  |  | 267 |  |  |  | 801 (694; 925) | 100 (--) |
| **United States, Puerto Rico, Brazil, South Africa**  NCT00604175  (Kojic et al., 2014) | Phase II, open-label,  single-arm study with stratification by CD4+ cell count to assess the immunogenicity and safety of the 4vHPV vaccine. | 4vHPV  3 doses  (0, 2, 6M) | Women  13-45 | 319  67  56  56 | 7 | cLIA  (mMU/mL) | HPV16 |  | PP  98.5 (92.0; 100)  98.2 (90.4; 100)  92.9 (82.7; 98.0) |
|  |  |  |  | 78  71  61 |  |  | HPV18 |  | 91.0 (82.4; 96.3)  84.5 (74.0; 92.0)  75.4 (62.7; 85.5) |
|  | CD4 >350 cells/μL  CD4 201–350 cells/μL  CD4 ≤200 cells/μL |  |  | 73  63  64 | 7 | cLIA  (mMU/mL) | HPV16 | ITT  1200 (871; 1654)  1117 (746; 1672)  571 (328; 994) |  |
|  | CD4 >350 cells/μL  CD4 201–350 cells/μL  CD4 ≤200 cells/μL |  |  | 86  80  69 |  |  | HPV18 | 175 (126; 243)  171 (115; 255)  94 (59; 149) |  |

| **Location**  **Clinical trial No.**  **(Reference)** | **Description** | **Vaccine product**  **No. Doses**  **(schedule)** | **Population**  **Age (years)** | **Sample size** | **Timing of measure**  **(months)** | **Results** | | | |
| --- | --- | --- | --- | --- | --- | --- | --- | --- | --- |
| **Immunogenicity endpoint** | | | | | | **Assay** | **HPV type** | **GMT (95% CI)** | **Seroconversion**  **% (95% CI)** |
| **United States, Puerto Rico, Brazil, South Africa**  NCT00604175  (Cespedes et al., 2018) | Sustained seropositivity  CD4 >350 cells/μL  CD4 201–350 cells/μL  CD4 ≤200 cells/μL |  |  |  | 18 | cLIA  (mMU/mL) | HPV16 |  | 95.2 (86.5; 99.0) 87.7 (76.3; 94.9)  85.5 (73.3; 93.5) |
|  | CD4 >350 cells/μL  CD4 201–350 cells/μL  CD4 ≤200 cells/μL |  |  |  |  |  | HPV18 |  | 69.1 (56.7; 79.8)  71.7 (58.6; 82.5) 53.2 (38.1; 67.9) |
|  | CD4 >350 cells/μL  CD4 201–350 cells/μL  CD4 ≤200 cells/μL |  |  | 63  59  64 | 18 | cLIA  (mMU/mL) | HPV16 | 249 (171; 362)  170 (107; 272)  98 (62; 156) |  |
|  | CD4 >350 cells/μL  CD4 201–350 cells/μL  CD4 ≤200 cells/μL |  |  | 75  73  69 |  |  | HPV18 | 42 (30; 60)  41 (27; 61)  21 (15; 30) |  |
| **South Africa**  NCT00586339  (Denny et al., 2013) | Phase I/II, partially blind, partially randomized, placebo-controlled trial of 2vHPV vaccine in HIV-positive women. | 2vHPV  3 doses  (0, 1, 6M)  vs. placebo | Women  18-25 | 150  120 HIV+  30 HIV- | Mo 7& M12 | ELISA  (EU/ml) | HPV16  HPV18 |  | 100  100 |
|  | HIV+  HIV- |  |  |  | 7 |  | HPV16 | 3558 (2724; 4649)  8169 (6341; 10,524) |  |
|  | HIV+  HIV- |  |  |  |  |  | HPV18 | 1946 (1451; 2609)  3703 (2503; 5479) |  |
|  | HIV+  HIV- |  |  |  | 12 |  | HPV16 | 748 (520; 1076)  2794 (2088; 3738) |  |
|  | HIV+  HIV- |  |  |  |  |  | HPV18 | 343 (236; 498)  1021 (627; 1663) |  |

| **Location**  **Clinical trial No.**  **(Reference)** | **Description** | **Vaccine product**  **No. Doses**  **(schedule)** | **Population**  **Age (years)** | **Sample size** | **Timing of measure**  **(months)** | **HPV type** | **Results** |
| --- | --- | --- | --- | --- | --- | --- | --- |
| **Other endpoints** | | | | | | | |
| **Canada**  (McClymont et al., 2019) | Cohort study to evaluate vaccine failure of the 4vHPV among participants of an open-label, multicenter, single-arm study evaluating the immunogenicity and safety of the 4vHPV vaccine among women and girls with HIV (ISRCTN33674451) | 4vHPV  3 doses  (0, 2, 6M) | Women and girls with HIV  9-65 | 420  Efficacy cohort:  n=212/4 cases  n=211/4 cases  n=177/0 cases | 24 |  | Incident rate per 100 person-years  Per-protocol efficacy population  Breakthrough persistent infections: 1.0 (0.3; 2.6)  Genital warts: 1.0 (0.3; 2.5)  CIN2+: 0 (0.0; 1.1) |
|  |  |  |  | n=268/11 cases  n=264/11 cases  n=217/0 cases | 24 |  | ITT population  Breakthrough persistent infections: 2.3 (1.1; 4.1)  Genital warts: 2.3 (1.2; 4.1)  CIN2+: 0 (0.0; 0.9) |
|  | Vaccinated population: WWH vs. HIV- |  |  |  |  |  | Rate ratios of infection and disease  Per-protocol efficacy:  11.7 (2.6; 52.1) |
|  | Vaccinated WWH vs. placebo HIV- |  |  |  |  |  | 0.8 (0.2; 2.5) |
|  | Vaccinated population: WWH vs. HIV- |  |  |  |  |  | ITT  1.1 (0.6; 2.2) |
|  | Vaccinated WWH vs. placebo HIV- |  |  |  |  |  | 0.8 (0.4; 1.5) |
| **Denmark**  (Zurek Munk-Madsen et al., 2018) | Phase IV, randomized, double-blind clinical trial to compare the immunogenicity and reactogenicity of 2vHPV and 4vHPV in HIV-infected adult men and women (NCT01386164) to determine the cellular immunity to HPV vaccines in HIV-infected, ART-treated men and women. |  |  |  |  |  | HPV vaccine-specific CD4/CD154/IL-2 positive T cells  T cells median frequency (IQR) |
|  |  | 4vHPV  (0, 1.5, 6M) | HIV+ men and women  ≥18 | 15 | 0  7  12 | HPV16 L1 | 0.005 (0.000–0.030)  0.040 (0.020–0.100)**  0.050 (0.020–0.070)** |
|  |  | 2vHPV  (0, 1.5, 6M) |  | 15 | 0  7  12 |  | 0.010 (0.000–0.020)  0.050 (0.010–0.113)**  0.030 (0.010–0.050)** |
|  |  | 4vHPV  (0, 1.5, 6M) |  | 15 | 0  7  12 | HPV18 L1 | 0.000 (0.000–0.003)  0.030 (0.010–0.040)**  0.020 (0.010–0.050)** |
|  |  | 2vHPV  (0, 1.5, 6M) |  | 15 | 0  7  12 |  | 0.000 (0.000–0.005)  0.040 (0.010–0.070)**  0.020 (0.000–0.060) |

| **Location**  **Clinical trial No.**  **(Reference)** | **Description** | **Vaccine product**  **No. Doses**  **(schedule)** | **Population**  **Age (years)** | **Sample size** | **Timing of measure**  **(months)** | **HPV type** | **Results** |
| --- | --- | --- | --- | --- | --- | --- | --- |
| **Other endpoints** | | | | | | | |
| **United States,**  **Puerto Rico**  (Weinberg et al., 2018) | Evaluation of additional potential protective mechanisms by investigating the persistence of memory B and T cell responses to 4HPV for 4 to 5 years after the last vaccine dose among HIV-1 infected children previously enrolled in IMPAACT P1047. (IMPAACT P1085; NCT01206556) |  |  |  |  |  | IFN T cell responses [median (Q1-Q3)] |
|  |  | 4 doses  (0, 2, 6, 24M) | Children  7-11 | 54 | 24  42  48-60 | HPV16 vs. HPV18 | 5 (SFC/105PBMC) (2-17) vs. 3 (2-9)**  5 (3-10) vs. 4 (2-8)**  4 (1-12) vs. 3 (1-9)** |
|  |  | 3-doses  (0, 2, 6M –initial placebo group) |  | 12 | 24  42  48-60 | HPV16 vs. HPV18 | 5 (4-15) vs. 2 (1-3)**  2 (1-8) vs. 2 (1-6)  1 (1-2) vs. 2 (0-5)** |
|  |  |  |  |  |  |  | IL2 T cell responses [median (Q1-Q3)] |
|  |  | 4 doses  (0, 2, 6, 24M) |  | 54 | 24  42  48-60 | HPV16 vs. HPV18 | 28 (SFC/105PBMC) (8-59) vs. 15 (6-38)**  25 (7-50) vs. 13 (5-45)**  17 (2-36) vs. 17 (3-33) |
|  |  | 3 doses  (0, 2, 6M –initial placebo group) |  | 12 | 24  42  48-60 | HPV16 vs. HPV18 | 16 (8-46) vs. 9 (2-23)**  12 (2-48) vs. 19 (1-40)  3 (1-13) vs. 2 (0-10)** |

Abbreviations: 2vHPV, bivalent HPV vaccine (Cervarix; GlaxoSmithKline); 4vHPV quadrivalent HPV vaccine (Gardasil; Merck & Co.); 9vHPV nonavalent HPV vaccine (GARDASIL 9, Merck & Co.); ART, Antiretroviral therapy; ATP, According to protocol; CI, confidence interval; CIN, cervical intraepithelial neoplasia; cLIA, competitive Luminex immunoassay (milli-Merck Units [mMU]/mL); ELISA, Enzyme-linked immunosorbent assay; GMT, geometric mean antibody titer; HAART, Highly active antiretroviral therapy; HIV, human immunodeficiency virus; HPV, human papillomavirus; HSIL, High-grade squamous intraepithelial lesions; IQR, Interquartile range; ITT, Intention-to-treat; LEEP, Loop electrosurgical excision procedure; M, Month; mITT, modified intention-to-treat (included all participants who received at least 1 study vaccine and all new persistent infections that occurred after the first vaccination); MSM, men who have sex with men; PBNA, Pseudovirion-based neutralization assay; PHEU, perinatally HIV-exposed, uninfected; PHIV, perinatally HIV-infected; PP, Per Protocol; PWH, people with HIV; SOT, Solid-organ transplant (performed at ≥12 months prior to the 1^st^ vaccination, did not have an acute rejection in the 6 months prior to the 1^st^ vaccination); TVC, Total vaccinated cohort; VE, Vaccine efficacy; WWH, women with HIV.

** Statistically significant

**References**

Boey, L., Curinckx, A., Roelants, M., Derdelinckx, I., Van Wijngaerden, E., De Munter, P., Vos, R., Kuypers, D., Van Cleemput, J., et al., 2021. **Immunogenicity and Safety of the 9-Valent Human Papillomavirus Vaccine in Solid Organ Transplant Recipients and Adults Infected With Human Immunodeficiency Virus (HIV)**. Clin Infect Dis 73:e661-e71. 10.1093/cid/ciaa1897.

Brophy, J., Bitnun, A., Alimenti, A., Lapointe, N., Samson, L., Read, S., Karatzios, C., Dobson, S., Moses, E., et al., 2018. **Immunogenicity and Safety of the Quadrivalent Human Papillomavirus Vaccine in Girls Living With HIV**. Pediatr Infect Dis J 37:595-97. 10.1097/inf.0000000000001874.

Cespedes, M.S., Kang, M., Kojic, E.M., Umbleja, T., Godfrey, C., Webster-Cyriaque, J.Y., Masih, R., Firnhaber, C., Grinsztejn, B., et al., 2018. **Anogenital human papillomavirus virus DNA and sustained response to the quadrivalent HPV vaccine in women living with HIV-1**. Papillomavirus Res 6:15-21. 10.1016/j.pvr.2018.08.002.

Denny, L., Hendricks, B., Gordon, C., Thomas, F., Hezareh, M., Dobbelaere, K., Durand, C., Hervé, C., Descamps, D., 2013. **Safety and immunogenicity of the HPV-16/18 AS04-adjuvanted vaccine in HIV-positive women in South Africa: a partially-blind randomised placebo-controlled study**. Vaccine 31:5745-53. 10.1016/j.vaccine.2013.09.032.

Firnhaber, C., Swarts, A., Jezile, V., Mulongo, M., Goeieman, B., Williams, S., Faesen, M., Michelow, P., Wilkin, T., 2021. **Human Papillomavirus Vaccination Prior to Loop Electroexcision Procedure Does Not Prevent Recurrent Cervical High-grade Squamous Intraepithelial Lesions in Women Living With Human Immunodeficiency Virus: A Randomized, Double-blind, Placebo-controlled Trial**. Clin Infect Dis 73:e2211-e16. 10.1093/cid/ciaa1456.

Folschweiller, N., Teixeira, J., Joshi, S., Goldani, L.Z., Supparatpinyo, K., Basu, P., Chotpitayasunondh, T., Chetchotisakd, P., Ruxrungtham, K., et al., 2020. **Immunogenicity and safety of the AS04-HPV-16/18 and HPV-6/11/16/18 human papillomavirus vaccines in asymptomatic young women living with HIV aged 15-25 years: A phase IV randomized comparative study**. EClinicalMedicine 23:100353. 10.1016/j.eclinm.2020.100353.

Kahn, J.A., Xu, J., Kapogiannis, B.G., Rudy, B., Gonin, R., Liu, N., Wilson, C.M., Worrell, C., Squires, K.E., 2013. **Immunogenicity and safety of the human papillomavirus 6, 11, 16, 18 vaccine in HIV-infected young women**. Clin Infect Dis 57:735-44. 10.1093/cid/cit319.

Kojic, E.M., Kang, M., Cespedes, M.S., Umbleja, T., Godfrey, C., Allen, R.T., Firnhaber, C., Grinsztejn, B., Palefsky, J.M., et al., 2014. **Immunogenicity and safety of the quadrivalent human papillomavirus vaccine in HIV-1-infected women**. Clin Infect Dis 59:127-35. 10.1093/cid/ciu238.

Levin, M.J., Moscicki, A.B., Song, L.Y., Fenton, T., Meyer, W.A., 3rd, Read, J.S., Handelsman, E.L., Nowak, B., Sattler, C.A., et al., 2010. **Safety and immunogenicity of a quadrivalent human papillomavirus (types 6, 11, 16, and 18) vaccine in HIV-infected children 7 to 12 years old**. J Acquir Immune Defic Syndr 55:197-204. 10.1097/QAI.0b013e3181de8d26.

Levin, M.J., Huang, S., Moscicki, A.B., Song, L.Y., Read, J.S., Meyer, W.A., Saah, A.J., Richardson, K., Weinberg, A., 2017. **Four-year persistence of type-specific immunity after quadrivalent human papillomavirus vaccination in HIV-infected children: Effect of a fourth dose of vaccine**. Vaccine 35:1712-20. 10.1016/j.vaccine.2017.02.021.

McClymont, E., Lee, M., Raboud, J., Coutlée, F., Walmsley, S., Lipsky, N., Loutfy, M., Trottier, S., Smaill, F., et al., 2019. **The Efficacy of the Quadrivalent Human Papillomavirus Vaccine in Girls and Women Living With Human Immunodeficiency Virus**. Clin Infect Dis 68:788-94. 10.1093/cid/ciy575.

Money, D.M., Moses, E., Blitz, S., Vandriel, S.M., Lipsky, N., Walmsley, S.L., Loutfy, M., Trottier, S., Smaill, F., et al., 2016. **HIV viral suppression results in higher antibody responses in HIV-positive women vaccinated with the quadrivalent human papillomavirus vaccine**. Vaccine 34:4799-806. 10.1016/j.vaccine.2016.08.016.

Moscicki, A.B., Karalius, B., Tassiopoulos, K., Yao, T.J., Jacobson, D.L., Patel, K., Purswani, M., Seage, G.R., 2019. **Human Papillomavirus Antibody Levels and Quadrivalent Vaccine Clinical Effectiveness in Perinatally Human Immunodeficiency Virus-infected and Exposed, Uninfected Youth**. Clin Infect Dis 69:1183-91. 10.1093/cid/ciy1040.

Mugo, N., Eckert, L.O., Odero, L., Gakuo, S., Ngure, K., Celum, C., Baeten, J.M., Barnabas, R.V., Wald, A., 2021. **Antibody responses to prophylactic quadrivalent human papillomavirus vaccine at 48 months among HIV-infected girls and boys ages 9-14 in Kenya, Africa**. Vaccine 39:4751-58. 10.1016/j.vaccine.2020.12.020.

Mugo, N.R., Eckert, L., Magaret, A.S., Cheng, A., Mwaniki, L., Ngure, K., Celum, C., Baeten, J.M., Galloway, D.A., et al., 2018. **Quadrivalent HPV vaccine in HIV-1-infected early adolescent girls and boys in Kenya: Month 7 and 12 post vaccine immunogenicity and correlation with immune status**. Vaccine 36:7025-32. 10.1016/j.vaccine.2018.09.059.

Palefsky, J., Poongulali, S., Lensing, S., Lee, J., Da Costa, M., Jeeva, A., Iqbal, S., Kumarsami, N., 2014. **AMC 054: safety and immunogenicity of the quadrivalent HPV vaccine in Indian HIV-infected women.** Boston: Conference on Retroviruses and Opportunistic Infections, p. 2014.

Palefsky, J.M., Poongulali, S., Lensing, S., Lee, J., Da Costa, M., Chein, A., Beulah, F., Murugavel, K.G., Kumarasamy, N., 2021. **AIDS Malignancy Consortium 054: Safety and Immunogenicity of the Quadrivalent Vaccine in Indian Women Living With HIV**. J Acquir Immune Defic Syndr 87:875-81. 10.1097/qai.0000000000002657.

Staadegaard, L., Rönn, M.M., Soni, N., Bellerose, M.E., Bloem, P., Brisson, M., Maheu-Giroux, M., Barnabas, R.V., Drolet, M., et al., 2022. **Immunogenicity, safety, and efficacy of the HPV vaccines among people living with HIV: A systematic review and meta-analysis**. EClinicalMedicine 52:101585. 10.1016/j.eclinm.2022.101585.

Toft, L., Storgaard, M., Müller, M., Sehr, P., Bonde, J., Tolstrup, M., Østergaard, L., Søgaard, O.S., 2014. **Comparison of the immunogenicity and reactogenicity of Cervarix and Gardasil human papillomavirus vaccines in HIV-infected adults: a randomized, double-blind clinical trial**. J Infect Dis 209:1165-73. 10.1093/infdis/jit657.

Weinberg, A., Song, L.Y., Saah, A., Brown, M., Moscicki, A.B., Meyer, W.A., 3rd, Bryan, J., Levin, M.J., 2012. **Humoral, mucosal, and cell-mediated immunity against vaccine and nonvaccine genotypes after administration of quadrivalent human papillomavirus vaccine to HIV-infected children**. J Infect Dis 206:1309-18. 10.1093/infdis/jis489.

Weinberg, A., Huang, S., Moscicki, A.B., Saah, A., Levin, M.J., 2018. **Persistence of memory B-cell and T-cell responses to the quadrivalent HPV vaccine in HIV-infected children**. Aids 32:851-60. 10.1097/qad.0000000000001773.

Wilkin, T.J., Chen, H., Cespedes, M.S., Leon-Cruz, J.T., Godfrey, C., Chiao, E.Y., Bastow, B., Webster-Cyriaque, J., Feng, Q., et al., 2018. **A Randomized, Placebo-Controlled Trial of the Quadrivalent Human Papillomavirus Vaccine in Human Immunodeficiency Virus-Infected Adults Aged 27 Years or Older: AIDS Clinical Trials Group Protocol A5298**. Clin Infect Dis 67:1339-46. 10.1093/cid/ciy274.

Zurek Munk-Madsen, M., Toft, L., Kube, T., Richter, R., Ostergaard, L., Søgaard, O.S., Tolstrup, M., Kaufmann, A.M., 2018. **Cellular immunogenicity of human papillomavirus vaccines Cervarix and Gardasil in adults with HIV infection**. Hum Vaccin Immunother 14:909-16. 10.1080/21645515.2017.1407896.
